# Supplementary material for: Are carbon emissions trading and green financial instruments synergistic? -Comprehensive quantitative research based on content analysis
Source: PLoS One. 2024 Mar 7;19(3):e0298601. doi: 10.1371/journal.pone.0298601 (PMC10919601; doi:10.1371/journal.pone.0298601)
Supplement: S1 Data — (ZIP) [file pone.0298601.s001.zip › Data/地级市Z_F工作报告.zip!/╣π╢1⁄2/╜╥╤⌠╩╨/└┤╫╘╣1⁄2╓┌║┼í╛┬φ┐╦╩2╛▌═°í┐_2007.docx]

各位代表：

　　我代表市人民政府向大会作政府工作报告，请予审议，并请政协各位委员和其他列席人员提出意见。

　　过去四年的工作回顾

　　本届政府自2003年6月组成以来，在省委、省政府和市委的正确领导下，在市人大及其常委会和市政协的监督支持下，坚持以邓小平理论和“三个代表”重要思想为指导，以科学发展观统揽全局，以抢抓机遇、加快发展为主题，采取强力措施，狠抓工作落实，促进全市经济社会持续较快健康发展。预计2006年，生产总值488亿元，比上年增长14.88%，四年年均（下同）增长9.3%；产业结构逐步优化，三次产业比例由2002年的22.1:45.1:32.8调整为15.3:49.9:34.8。

　　——生产持续较快发展，质量效益逐步提高

　　工业经济增长较快。纺织服装、五金机械、化工塑料、食品医药和电子信息等五大产业不断发展壮大，产业集群逐步形成。巨轮模具、运通塑料2家企业成功上市，累计上市企业达到6家。新增规模以上工业企业337家，累计791家；新增中国名牌产品2个、国家免检产品6个、广东省名牌产品15个、驰名商标2件、著名商标32件，累计分别达到2个、7个、18个、2件和46件；先后荣获“亚洲玉都” 、“中国玉都” 和“中国五金基地”等称号，普宁市、惠来县分别荣获“中国纺织产业基地”和“中国能源工业大县”称号。揭阳（惠来）沿海经济开发试验区（揭阳石化综合工业区）、揭阳高新技术产业开发区、揭阳大南山工业区前期工作进展顺利。2006年，规模以上工业总产值377亿元，增长24%，年均增长20.5%；工业经济效益综合指数150%，比上年提高8.3个百分点，比2002年提高47.85个百分点。

　　农业生产稳步发展。基本农田保护得到加强，粮食生产保持稳定。无公害、绿色、有机农产品基地建设有效推进，效益农业发展进一步加快。6大农业支柱产业基本形成，12大农业商品基地建设规模不断扩大。普宁蕉柑、揭东竹笋被列为国家地理标志保护产品，11个国家农业综合开发项目、1个国家柑桔良种无病毒苗木繁育中心、2个省级农业现代化示范功能区和41个市级以上标准化示范区建设顺利推进，9个省级、26个市级农业地方标准得到推广应用。18家省、市级农业龙头企业不断发展壮大，农业产业化进程加快。2006年，农林牧渔业总产值116亿元，增长2.7%，年均增长3.8%。

　　——投资建设有效推进，发展环境不断优化

　　基础建设取得成效。揭普、梅揭高速公路建成通车，潮揭和汕揭高速公路揭阳段、市区南河大桥、西凤大桥扩建新建工程加快推进，潮汕民用机场、厦深铁路揭阳段建设前期工作进展顺利。惠来电厂1、2号机组即将建成发电，石碑山风电场基本竣工投产；500千伏榕江和220千伏华湖、紫峰、铁山等输变电工程建设步伐加快；惠来大型核电、LNG项目前期工作正在抓紧推进。地都、东陇堤防建设及达标加固顺利实施，汤坑、石榴潭等水库除险加固工程基本完成，城乡防灾减灾能力不断增强。2006年，全社会固定资产投资152亿元，增长30.8%，四年累计441.57亿元，年均增长17.9%。

　　城乡建设步伐加快。引资30亿元建设市区城市设防综合工程及配套市政设施顺利实施，黄岐山大道改造和临江北路、建阳路、美阳路、新阳路等市政道路路面修复及绿化配套基本完成，市垃圾处理场首期工程、榕江大型音乐喷泉等重点项目建成投入使用，国道206线市区至新亨互通立交、市区污水处理厂等项目建设加快推进。各县（市、区）每年筹资1亿元以上投入城区市政公共设施建设任务得到落实，城区市政功能逐步配套。镇级规划编制工作基本完成，社会主义新农村村庄规划编制正在抓紧进行，71个新农村示范点建设顺利推进，新农村建设正在逐步铺开。

　　——市场流通日趋畅旺，内外经贸成效明显

消费需求有效扩大。积极组织参加广交会、珠洽会、农博会、中博会、山洽会等各类经贸洽谈会，成功举办了4届玉器节、衬衣节暨纺织服装交易会以及揭阳轻工产品（泰国）展销会、五金不锈钢制品博览会、揭西生态旅游文化节等节会，会展经济蓬勃发展。金属材料市场、粮食市场、揭阳卷烟配送中心和普宁粤东卷烟物流配送中心等建设顺利推进，粤东鞋材城、水果批发市场、五金制品交易中心和普宁轻纺城等市场逐步配套完善。旅游、汽车、通讯、商住等消费逐步形成热点，物流、物业、社区、中介等现代服务业稳步发展。2006年，社会消费品零售总额167.31亿元，增长16.7%，年均增长13.4%。

　　对外经贸快速发展。新增外贸进出口经营权企业268家，累计416家。纺织服装、鞋类、不锈钢制品等传统出口产品和机电产品、高新技术产品出口增长较快，出口结构逐步优化。2006年，外贸出口总额13.36亿美元，增长18%，年均增长31.8%。招商引资力度加大，成功举办中国经济年度论坛暨亚洲企业领袖（揭阳）年会和2场招商引资大会，引进百事可乐、泰国正大集团2家世界500强企业在我市设立生产基地或连锁经营企业。四年累计实施外引内联项目354个，实际引进境内市外资金176.22亿元；新签利用外资合同项目199个，合同利用外资6.54亿美元，实际利用外资3.7亿美元。

　　——财税金融运行平稳，人民生活有所改善

　　财税金融管理得到加强。市、县、镇三级财政管理体制进一步调整理顺，税收增长激励机制和联动协税网络逐步建立健全。部门预算、政府采购、国库集中支付等制度改革有效实施，公共财政体制框架初步形成。财政资金追收和金融资产清收力度加大，财政金融风险逐步得到化解。农信社改革试点工作顺利铺开，地方金融秩序保持稳定。2006年，地方财政一般预算收入13.63亿元，增长21.8%，年均增长10.21%；其中工商税收9.24亿元，增长25.02%，年均增长20.32%。

　　人民生活水平逐步提高。城乡统筹就业有效推进，四年累计新增就业岗位6.9万个，城镇登记失业率控制在3.5%以内。社保扩面力度加大，征缴机制逐步完善。社会保险费累计收入16.54亿元，年均增长37.6%；其中，企业养老保险金收入6.91亿元，年均增长40.4%。国企下岗职工基本生活保障和城乡居民最低生活保障制度得到落实，养老、医疗、失业、工伤等保险制度顺利实施，社会保障体系逐步建立健全。2006年，城镇居民人均可支配收入9860元，增长7.3%，年均增长9.1%；农民人均纯收入4291元，增长4%，年均增长3.7%。

　　——各项事业协调推进，社会保持和谐稳定

　　社会事业全面进步。教育事业发展加快。农村义务教育免收杂费政策得到落实，“普九”成果得到巩固提高。四年累计投入校舍建设资金16.5亿元，新扩建中小学312所，改造老区小学341所；揭阳一中新校区、市技工学校新校区首期工程建成投入使用，揭阳侨中新校区主体工程即将竣工。师生素质得到有效提高，共增配教师7242人；2006年每万人口在校普通高中生153人，比2002年末增加77人。科技工作取得成效。实施国家和省各类科技计划207项，新增国家、省级高新技术企业26家、省级民营科技企业44家、省级专业镇8个，新设立国家级企业技术中心1个、省级工程技术研发中心2个、博士后工作站3个。公共卫生得到加强。抗击非典和禽流感疫情取得重大胜利。公共卫生应急体系不断完善，卫生服务网络逐步健全。农村合作医疗制度有效实施，新型农村合作医疗农业人口覆盖率32%。其他事业扎实推进。文化遗产和历史名城保护力度加大，文艺创作事业不断繁荣发展。全市1445个行政村广播站建设已全面完成，实现“村村通广播”目标。与泰国南邦市成功结为友好城市，与华工大、华农大合作有序推进。经济普查工作顺利完成。体育、物价、统计、气象、地震、人民防空、国防动员、民族宗教、文史档案、广播电视、新闻出版等事业得到发展。

各项管理力度加大。人口与计生管理不断加强，人口增长得到有效控制，完成省下达的目标任务。殡改成果得到巩固，火化率保持100%。环境保护力度加大，四年共投入整改治理资金3亿多元，清理整治违法排污企业997家，其中依法取缔、关闭562家；榕江、练江、龙江、枫江等重点流域水污染得到有效遏制。土地、矿产、海洋等资源保护力度加大，林业建设得到加强，森林覆盖率达49%。偷税漏税、制假售假、走私贩私、非法传销等违法犯罪活动受到严厉打击，土地、建筑、文化、食品、药品等市场秩序进一步规范。交通运输、危险化学品等专项整治扎实开展，安全生产形势保持稳定。以打黑除恶和打击“两抢一盗”、绑架勒索、赌博等为重点的严打整治斗争深入开展，各种刑事犯罪和社会丑恶现象受到打击。打击“法轮功”邪教组织工作取得成效。“禁毒03工程”扎实推进，普宁“毒品重灾区”帽子顺利摘除。基层调解、信访工作继续加强，突发性事件、群体性事件得到妥善处置，社会局面保持稳定。

　　——民主法制进程加快，自身建设不断加强

　　民主法制建设扎实推进。定期向人大及其常委会报告工作、向政协通报情况制度进一步完善，人大及其常委会的决议、决定得到认真执行。与工、青、妇等群团组织的联系更加紧密，社会各界的意见建议得到重视和吸纳，政府决策的科学化、民主化水平不断提高。四年共办理市人大代表建议、议案236件，政协委员提案、建议意见323件。基层民主政权建设加快推进，县、镇两级换届顺利完成，村（居）民自治制度逐步完善，政务、村务、厂务公开顺利实施。“四五”普法任务圆满完成，“五五”普法规划顺利实施，全民法制观念不断增强。

　　政府自身建设得到加强。行政许可法等法律法规得到有效实施，行政审批制度改革不断深化，完成第二、三轮行政审批制度改革。四年共制定政府规范性文件33件，废止涉及行政许可规定的政府性文件22个，取消和调整审批项目613项。政府制度建设质量有所提高，行政执法监督力度进一步强化。审计监督成效明显，受到国家表彰。政府五项廉政承诺有效履行，行业不正之风得到纠正，人民群众反映强烈的热点问题得到有效解决。机关效能建设扎实推进，公务员队伍建设得到加强，行政效率和服务水平逐步提高。

　　各位代表，过去四年，我市经济社会保持较快发展，取得了较好的成绩。这是省委省政府和市委正确领导的结果，是各级人大及其常委会和政协监督支持的结果，是全市人民团结拼搏、共同努力的结果，是中央和省驻揭单位、驻揭人民解放军、武警官兵、各民主党派、人民团体、港澳台同胞、海外侨胞、海内外乡亲和友好人士关心支持的结果。在此，我谨代表市人民政府表示衷心的感谢并致以崇高的敬意！

　　回顾过去四年的工作，我们也清醒地看到，我市经济社会发展仍存在不少困难和问题。突出表现在：经济增长质量偏低，总体水平不高；产业结构不够合理，发展基础仍较薄弱；企业普遍“小、弱、散”，自主创新能力不强；发展环境有待改善，投资拉动仍较乏力；县域经济发展滞后，城镇化进程缓慢；村镇经济仍较薄弱，债务负担较重；市场秩序有待深入整顿和规范，环境污染治理任务繁重；治安形势仍较严峻，维护稳定压力较大；干部队伍素质有待提高，机关作风建设尚需进一步加强。对此，我们要采取更加有力的措施，在加快发展中认真解决。

　　今后五年的奋斗目标和主要任务

　　今后五年，是我市贯彻粤东会议精神、全面建设小康社会的关键时期，是打基础上台阶、强势推进跨越发展的攻坚阶段。刚刚召开的市第四次党代会为我们描绘了未来五年的发展蓝图，明确提出建设富民强市、和谐文明新揭阳的奋斗目标。实现这个宏伟目标，任务艰巨，责任重大。我们必须坚持科学发展不动摇，咬定发展目标不放松，振奋精神，坚定信心，树立“一代而强”的英雄气概，凝心聚力促发展，众志成城兴揭阳，为实现经济社会大发展打下坚实的基础。

今后五年，市政府工作的指导思想：以邓小平理论和“三个代表”重要思想为指导，以科学发展观统领经济社会发展全局，认真贯彻落实中央十六届六中全会和省委九届九次、十次全会以及市第四次党代会精神，按照“三年打基础、五年大变化、十年大发展”的总要求，围绕建设富民强市、和谐文明新揭阳的总目标，狠抓发展第一要务，加快实施工业强市、文化大市、商贸旺市、三农优先、绿色揭阳“五大战略”，强势推进工业发展、城镇建设、基础教育“三个突破”，全力加快经济发展，协调推进社会事业，加强民主法制建设，保持社会和谐安定，努力实现经济社会科学、健康、跨越发展。

　　今后五年，我市经济社会发展的主要预期目标：生产总值年均增长21.2%，单位生产总值能耗年均下降3.43%，工业增加值年均增长23.6%，农林牧渔业总产值年均增长4.5%，全社会固定资产投资年均增长30%，社会消费品零售总额年均增长19.5%，外贸出口年均增长21.4%，实际利用外资年均增长44%，地方财政一般预算收入年均增长25.11%，力争到2011年，经济总量比2006年翻一番半以上，三次产业结构调整为8.1∶53.5∶38.4，经济发展进入快车道，基本形成产业有效集聚、功能比较配套、规模效益明显的粤东制造业基地和物流中心；各项事业全面协调发展，社会文明程度不断提高，发展环境得到有效改善，基本形成开放兼容、诚信友爱、文明法治、稳定和谐的社会环境；城乡面貌明显改观，市区核心区域辐射和带动作用显著增强，基本建成富有水乡特色的粤东明珠城市。

　　实现上述目标，必须进一步转变发展观念，创新思维方式，改进工作方法，做到“五个坚持”：坚持科学发展，加快增长方式转变，在提高质量中加快发展，在加快发展中提高质量和效益，促进经济社会又好又快发展；坚持改革开放，加快推进体制机制创新，以大改革推动大开放，以大开放促进大招商，以大招商推动大发展；坚持以人为本，始终把最广大人民的根本利益作为一切工作的出发点和落脚点，大力发展各项社会事业，不断满足人民日益增长的物质文化需要，让发展成果惠及全体人民；坚持和谐稳定，统筹协调各方面利益关系，妥善处置各种社会矛盾，保持社会和谐安定；坚持民主法治，发展社会主义民主，增强全民法律意识，加快政府职能转变，不断提高政府驾驭市场经济、构建和谐社会、应对复杂情况和处置突发事件的能力。为此，要着力抓好五方面工作：

　　一、着力提升经济素质，加快产业优化升级

　　以调整优化经济结构为主线，切实提高经济增长质量和效益，推动三大产业优化升级。发展壮大第二产业。鼓励运用先进适用技术改造提升传统产业，大力发展能源、石油化工以及电子信息等高新技术产业，培植壮大产业集群。积极引导扶持优秀骨干企业拓宽融资渠道，走资本化经营道路，发展成为上市公司。大力培植规模以上企业，着力壮大企业规模，不断做大做强工业经济。搞活搞旺第三产业。转变外贸增长方式，调整优化进出口结构，加快发展对外贸易。积极参加各类经贸洽谈会，认真办好地方特色产品节会，努力开拓国内市场。加强市场规划建设管理，大力发展现代服务业和旅游业，有效扩大消费需求。巩固提高第一产业。调整优化农业种养结构，大力发展效益农业、特色农业和生态农业，提高农业综合效益。巩固发展农业支柱产业，推进农业商品基地和特色水产养殖基地建设。加强农业标准制订和推广应用，提高农产品质量安全水平。培植壮大农业龙头企业，加快农产品市场建设，健全农业社会化服务体系，推进农业产业化进程。

　　二、着力推进投资建设，加快改善发展环境

　　加大投资建设力度，加快交通、能源、水利、通信等基础设施建设，为加快发展提供良好环境。多方筹措建设资金。深化投融资体制改革，进一步放开投资领域，大胆创新筹资机制，大力拓宽融资渠道，有效利用民资、外资和侨资，争取国债资金、上级专项补助和银信资金支持，多渠道多形式筹集建设资金。狠抓重大项目引进。切实搞好重大项目策划，积极主动搞好衔接跟进，争取更多重大项目在我市落户。认真研究利用上级政策，精心谋划和筛选一批符合国家产业政策的重点项目，积极组织向上申报，争取列入国家和省投资建设的“笼子”。加快基础设施建设。加快实施能源、交通、水利、环保、文化“五大工程”，尽快构建布局科学、适度超前、综合配套、适应快速发展需要的基础设施体系。

　三、着力深化改革开放，加快体制机制创新

　　继续推进各项改革，扩大对内对外开放，加强区域交流与合作，加快建立健全充满活力、富有效率、更加开放的体制机制。深化各项体制改革。积极推进国有企业改革，完善现代企业制度。健全国有资产监管体制，加强财务监督和管理。深化行政管理体制改革，加快政府职能转变。调整完善财政管理体制，建立健全公共财政体系。推进地方金融改革，完善中小企业贷款担保机制。健全现代市场体系，大力发展产权、资本、劳动力、技术等要素市场。努力提高开放水平。充分发挥侨乡优势，以扩大开放为突破口，加强对外联络和宣传推介，千方百计招商引资，大力吸引境内外资金和项目，推动内外源型经济共同发展，加快形成海纳百川、兼容并蓄的开放型经济体系。积极推进交流协作。加强与泰国南邦市合作与交流，推进与粤东各市协作，认真办好（或参与）“侨博会”，促进互利共赢发展。积极推进闽粤赣十三市区域经济协作，加强与“泛珠三角”、“大珠三角”区域的经贸联系与交流。认真实施与华工大、华农大合作协议，推动产学研合作。

　　四、着力壮大区域经济，加快城乡统筹发展

　　充分发挥各地资源优势和产业比较优势，认真实施“四大产业带”发展规划，加快形成分工合理、重点突出、特色明显的区域经济格局。大力发展特色经济。认真贯彻落实加快民营经济发展的优惠措施，大力发展地方特色产业，培育一批专、精、特的中小企业群，尽快形成各具特色、各有优势的产业集群，逐步发展壮大区域经济。全力推进市区发展。以引资30亿元建设市区城市设防综合工程及配套市政设施为突破口，加强市区规划、建设和经营管理，强力推进城市建设，加快构建区域核心，发挥龙头带动作用。加强城镇规划建设。抓紧督促各县（市、区）加强城区规划建设，加快中心镇基础设施、公共服务设施建设步伐，逐步完善城镇功能，促进城镇第二、三产业发展，增强辐射和带动农村的能力。加快新型农村建设。大力推广和落实玉湖先进经验，加强基层政权建设。全面推进农村综合配套改革，建立农民增收减负长效机制。加强农村规划建设，强化村容村貌综合整治，努力改善农村生产生活环境，建设社会主义新农村。

　　五、着力强化协调管理，加快构建和谐揭阳

　　按照建设社会主义和谐社会的要求，以解决人民群众最关心、最直接、最现实的利益问题为重点，大力发展各项事业，促进社会公平正义，着力打造平安和谐揭阳。协调发展社会事业。以提高市民文化素质为根本，优先发展教育事业。以增强自主创新能力为核心，积极推动科技进步。以建设文化大市为目标，大力发展和谐文化。协调推进卫生、体育等各项事业。高度关注民生民计。建立健全社会利益协调机制和收入分配调节机制，努力化解各种社会矛盾。切实关心群众生活，不断拓宽就业渠道，完善社会保障体系，积极帮扶弱势群体，着力解决群众切身利益问题。加强生态环境管理。强化人口、资源、环境综合管理，积极发展循环经济，优化生态环境，加快建设绿色揭阳，努力构建资源节约型、环境友好型、氛围和谐型社会，促进人与自然和谐相处。切实维护社会稳定。建立健全应急管理体制机制，有效应对自然灾害、公共卫生和社会安全事件，增强保障公共安全和处置突发事件的能力。继续整顿和规范市场经济秩序，严厉打击各种经济违法行为。加强安全生产监督管理，防止重特大安全事故的发生，确保人民群众生命财产安全。加强社会治安综合治理，依法严厉打击严重刑事犯罪活动，坚决扫除“黄赌毒”等社会丑恶现象，着力整治治安突出问题和重点地区，切实增强群众安全感。坚持依法行政，妥善处置各种社会矛盾，维护社会和谐稳定。

　　2007年的工作安排

　　2007年是全面贯彻落实粤东会议精神、促进经济社会快速发展的起步之年。今年我市经济社会发展的主要预期目标：生产总值增长18%，单位生产总值能耗下降3.43%，工业增加值增长21%，农林牧渔业总产值增长4%，全社会固定资产投资增长32%，社会消费品零售总额增长17%，外贸出口增长18%，实际利用外资增长30%，地方财政一般预算收入增长22.8%，城镇登记失业率控制在3.5%以内，人口自然增长率控制在7.2‰以下。

实现上述目标，必须狠抓十方面工作：

　　一、狠抓工业生产，壮大产业集群

　　做强一批主导产业。积极运用先进适用技术、高新技术和信息化手段，加快纺织服装、化工塑料、五金机械、食品医药等传统支柱产业和电子信息产业发展，大力引进和发展电力能源、石油化工、装备制造等新兴产业，积极推进中医药现代化，不断壮大工业实力。认真做好重点产业项目规划，大力发展模具、不锈钢制品等制造业，建设一批产业集群升级示范区，加强产业配套，延伸产业链条，加快培育形成特色产业集群。

　　培育一批龙头企业。进一步完善领导干部挂钩重点企业制度，认真落实扶持工业发展的优惠措施，从用地、融资、技术等方面加大扶持力度，培育形成一批龙头骨干企业。加强技术改造和创新，重点抓好榕泰年产6万吨氨基复合材料、康美中药饮片等技改项目实施工作。认真落实扶持民营经济做大做强的政策措施，积极实施30强民营企业重点培育计划，壮大民营经济实力。大力发展资本市场，鼓励龙头企业加快股份制改造，争取成功上市特别是成为境外上市公司。加大规模上企业培植力度，迅速壮大工业规模。

　　打造一批品牌产品。认真落实提高自主创新能力的各项配套措施，鼓励企业自主创新，大力支持企业申报品牌、商标和专利，积极培育和发展形成一批自主创新型品牌企业。力争新增国家免检产品1个、名牌产品6个、著名商标15件。

　　建设一批工业园区。在继续加强现有园区建设管理的同时，创新办园模式，转变办园主体，将园区建设管理由政府行为转变为企业行为，走市场化办园的新路子。认真搞好揭阳（惠来）沿海经济开发试验区（揭阳石化综合工业区）、揭阳高新技术产业开发区、空港经济区和普宁健康科技产业示范园等工业基地规划建设，为大办工业提供发展载体。加大揭阳大南山工业区的招商引资工作力度，争取创办东山承接产业转移园区，积极承接珠三角等发达地区产业转移。

　　二、狠抓城区建设，打造区域核心

　　严格规划开发。严格保护和合理开发市区58平方公里范围内存量3600亩土地、50平方公里新城区存量土地，依法依规坚决收回市区已出让但尚未建设的土地。认真搞好市区新城区的策划包装，引进素质高、实力强的开发商进行开发建设。抓紧抓好“金腰带”五千年中华历史文化景观长廊、临江北路2.7公里潮汕历史文化长廊规划设计，尽快组织实施，着力提升城市品位。加快市区旧城保护开发和城中村改造，严格加强县（市、区）城区土地控制和规划开发。

　　全力推进建设。加快实施引资30亿元建设市区城市设防综合工程及配套市政设施，确保按期有序推进。积极推进市行政服务中心、临江南路、揭阳楼等重点工程及配套市政设施建设，进一步完善城市功能。加强市区房地产市场管理，引导房地产开发商搞好规划设计，建设时尚高档和经济适用商住区，着力营造良好人居环境。继续抓紧督促各县（市、区）筹资1亿元以上投入城区市政公共设施建设，逐步完善城区功能，增强带动和辐射能力。

　　搞好经营管理。创新思路和方法，整合利用城市公共资源，对地下管网、广告经营权、市政设施冠名权等公用设施、城市无形资产实施市场化运营，提高经营城市水平。抓紧完善理顺市区管理体制和推进市政公用事业改革的政策措施，继续深入开展交通、环卫、绿化等专项整治，加大城市管理综合执法力度，综合治理“脏乱差噪”现象，树立良好城市形象。

　　三、狠抓重点项目，加强基础建设

　　加快能源建设。加快惠来电厂建设步伐，确保1、2号机组按期投产，争取3、4号机组尽快建设。继续做好惠来大型核电、LNG项目前期工作，力争早日落户我市。积极推进500千伏榕江变电站和220千伏铁山、紫峰等输变电工程以及惠来电厂送出线路工程建设，争取按期竣工投产。加快城乡电网配网建设和改造，满足城乡用电需求。

加快交通建设。着力推进潮汕民用机场征地拆迁和移民安置前期工作，力争今年下半年开工建设。积极配合推进厦深铁路和汕揭、潮揭高速公路揭阳段建设，加快国道206线揭丰段、省道236线池揭段改造和南河大桥扩建等工程建设步伐。积极推进汕湛和玉漳揭阳段、惠来至揭东等高速公路前期工作，认真开展榕江、靖海、神泉、南海等港口码头的规划。加快市通县、县通镇等级公路和通行政村公路硬底化建设，进一步完善公路网络。

　　加快水利建设。积极推进城乡防灾减灾工程建设，继续做好榕江大围打捆上报、三洲拦河闸申报核准工作，抓紧实施惠来“西水东调”计划；加快惠来赤岑水闸、揭东磐岭围、普宁流沙联围新河堤加固和惠来县城、揭西县城、金凤联围防洪工程等项目建设，提高城乡防灾减灾能力。

　　加快通信建设。抓紧建设全市统一电子政务网络平台，积极推进投资项目联网注册系统和行政审批电子监察网系统建设，推动电子商务建设。进一步完善公共信息网络基础设施，提高社会公共信息资源共享水平。

　　四、狠抓内外经贸，积极开拓市场

　　大力招商引资。认真做好招商项目的策划和包装，加强对外信息发布和宣传推介，提高招商引资的针对性和实效性。加强对外沟通联系，广泛动员在外揭籍企业家回乡投资创业，大力引进大企业、大财团特别是世界500强企业和跨国公司到我市落户。抓住招商引资大会、中国经济年度论坛暨亚洲企业领袖（揭阳）年会成功举办的契机，认真做好亚洲资本论坛永久会址等在谈项目跟踪引进，争取更多项目落户我市。切实搞好协调服务，确保金泓生态新城等签约项目顺利实施，努力提高项目履约率。

　　搞活商贸物流。积极组织企业参加广交会、珠洽会、农博会、中博会等各类经贸洽谈会，认真办好玉器、衬衣暨纺织服装、五金不锈钢制品、生态旅游文化等节会，大力发展会展经济。加快推进市区金属材料市场、东山商业文化步行街、水产品物流配送中心、普宁粤东卷烟物流配送中心和医药物流港建设，配套完善五金不锈钢制品交易中心、粤东鞋材城、市区汽贸一条街、市果菜批发市场、市茶叶批发市场、市卷烟配送中心和粮食市场等载体，搞活搞旺普宁服装市场、轻纺城、中药材市场等专业市场。继续实施“万村千乡”市场工程，进一步完善农村商品流通网络。大力发展连锁经营、现代物流、网上销售等新的流通业态，努力扩大消费需求。

　　拓展对外经贸。积极适应国际市场的发展变化，认真研究国家汇率和外贸政策，加快出口退税进度，培育壮大外贸经营主体，大力鼓励企业扩大出口。调整优化出口商品结构，提高出口的质量和效益。巩固港澳、欧美日、东盟等传统市场，多形式、多渠道开拓新兴国际市场。加强与海关、检验检疫、税务、外汇管理等部门的协调和沟通，营造良好通关环境，促进对外贸易快速发展。

　　五、狠抓“三农”工作，繁荣农村经济

　　调整种养结构。加强3个国家和省农业综合开发项目、1个国家柑桔良种无病毒苗木繁育中心以及3个省级农业现代化示范功能区建设，不断扩大规模化生产。加大良种良法引进、示范和推广力度，大力发展无公害、绿色、有机食品等优质农产品，逐步调整优化农业结构。加强动植物重大疫情防控，确保农业生产安全。积极发展现代渔业，提高海洋综合开发水平。推进科技兴林，加快林业产业化。大力扶持发展农产品加工企业，进一步培植壮大农业龙头企业，配套完善农产品市场，健全农民专业合作经济组织和农业服务体系，提高农业产业化水平。

　　建设新型农村。抓紧搞好新农村村庄建设规划编制，加快农村道路、电力、通讯、供水等公共设施建设，努力改善农村生产生活条件。大力整治村容村貌，切实改变农村环境“脏乱差”现象，建设生态和谐文明农村。认真抓好市、县、镇级71个示范点建设，总结经验，以点带面，逐步铺开社会主义新农村建设。

加强农村管理。稳定农村土地承包关系，推进集体建设用地使用权流转，盘活农村集体土地资产。加强农村集体经济组织建设，完善农村集体征地补偿款分配和使用的监督管理，坚决查处侵犯农民合法权益的违法行为。大力推广玉湖固本强基先进经验，积极推行村账镇管和审计监督，规范农村集体资产和财务管理。完善农村干部退休退职基金制度，不断改善和提高基层干部的政治待遇和生活待遇，调动基层干部工作积极性。

　　减轻农民负担。按照“多予少取放活”的方针，加大扶农惠农力度，加快农村集体经济发展。加强农民负担监管，防止农民负担反弹。积极实施“农村安居工程”，切实做好边远分散老区村庄搬迁工作，解决好贫困农民住房难问题。加大扶贫开发力度，认真落实扶持政策，加快贫困地区脱贫奔康步伐。

　　六、狠抓税费征管，增强财力保障

　　强力组织收入。大力培育壮大税源，坚持依法治税，加大税收征管力度，重点加强对增值税、营业税、企业所得税、个人所得税等重点税种的征收，把该收的税坚决收上来。强化税务稽查，严厉打击偷、漏、抗税等违法行为，确保应收尽收。加强对非税收入的征管，规范收费行为。落实地税全责征收机制，加大社保费征缴力度，扩大社会保险覆盖面。

　　强化支出管理。优化财政支出结构，在保运转、保稳定基础上，集中财力加大对重点工程和基础建设、农业、科教、社会保障、公共卫生等投入，扩大公共财政覆盖范围。完善国库集中支付制度，进一步规范零基预算、部门预算和综合预算编制管理，建设严格高效的财政支出管理体系。加强社保基金管理，做实个人帐户，增强保障能力。

　　加强财经监督。加快财政信息化建设，逐步建立实时在线财政预算监督系统。完善政府投资的监督管理机制，建立健全基建项目招投标、政府采购、派驻财务总监制度，推进财政支出绩效评价工作。强化财税监管、审计监督，严肃财经纪律，确保财政资金规范、安全、高效运行。加强地方金融监督管理，继续推进农信社改革试点工作。

　　七、狠抓各项事业，促进社会进步

　　优先发展教育。继续落实资金筹措、校舍建设和提高师生素质三大任务，强化工作责任考核，力促基础教育加快发展，全面提高教育教学质量。认真落实免费义务教育政策，巩固提高“普九”成果。大力发展普通高中教育，积极创建示范性高中。加快职业教育发展，认真办好市技校、综合中专等中等职业学校。扎实推进揭阳学院配套建设，力争今年底前通过国家达标评估。加快市卫校、揭阳侨中新校区建设，配套完善揭阳一中新校区，积极筹建市特殊教育学校。进一步推进教育人事制度改革，实行校长负责制，全面推行中小学校长竞争上岗、任期目标管理和绩效考评制度；加大教师招聘引进和学历达标教育培训力度，认真落实资助政策，鼓励教师报考北师大教育硕士，着力提高教师队伍整体素质。加强师生道德教育，完善学校、家庭、社会思想道德教育网络。

　　推动科技进步。引导和鼓励企业加大技术开发投入，发展壮大技术创新主体，健全科技创新服务体系。大力支持企业设立工程技术研发中心，积极扶持高新技术企业和民营科技企业，打造自主创新基地。力争新增6家高新技术企业、12家省级民营科技企业、1个省级工程技术研发中心。加强技术标准工作，依法保护知识产权。做好科普知识宣传，提高全民科技意识。

　　繁荣社会文化。认真实施文化大市建设规划，加强市文化中心、体育馆、档案馆、榕城文化广场、东湖公园等文化设施建设，逐步配套完善县、镇、村文化设施。大力发展地方特色文化，繁荣文艺事业。加强文物保护，做好揭阳学宫、丁氏光禄公祠和关帝庙等文化遗产申报国家级文保单位工作。建设文化产业园和文化产业基地，积极发展文化产业。

　　发展其他事业。进一步完善医疗救治体系和卫生信息网络，强化疾病预防控制和卫生监督。加快市人民医院、红十字会慈云医院住院大楼和市卫生监督所综合业务楼建设，力争尽快建成投入使用。加强农村医疗卫生服务体系建设，积极实施新型农村合作医疗制度，力争覆盖率达到70%以上。认真做好中国优秀旅游城市创建工作。积极发展妇女、儿童、老龄和残疾人事业。加强国防、民兵预备役和双拥工作，积极创建双拥模范城。继续推进体育、外事、侨务、台务、民族、宗教、统计、民政、物价、人防、气象、地震、新闻、广电、档案、方志、文史等各项事业。

八、狠抓民生民计，改善人民生活

　　统筹城乡就业。落实就业再就业政策，拓宽就业渠道，开发社区就业岗位和公益性岗位。落实小额担保贷款、税费减免等优惠政策，鼓励自主创业和自谋职业。积极实施“百万农村青年技能培训工程”、创业培训工程和退役士兵职业技能培训工程，加强就业培训和技工教育，提高劳动者素质和就业能力。

　　完善社会保障。深化医疗保险体制改革，建立健全覆盖养老、失业、工伤、医疗、生育保险的社会保障体系。认真落实城镇居民最低生活保障政策，逐步扩大农村低保覆盖面，提高低保水平。切实做好国企下岗职工解除劳动关系工作，积极稳妥解决国企改革遗留问题。支持发展慈善事业，建立经常性捐赠工作机制。加强城乡社会福利机构建设，努力发展社会福利事业。

　　关心群众生活。完善社会救助体系，加大扶贫助困力度。认真做好救灾救济工作，妥善安排好困难群众的生产生活。规范征地拆迁行为，切实保护群众合法权益。加大对困难家庭子女就学帮扶力度，确保不让一名学生因贫辍学。扎实推进改水工程建设，提高群众饮水安全水平。

　　九、狠抓各项管理，营造良好环境

　　优化人文环境。加强“八荣八耻”社会主义荣辱观教育，深入开展“爱国、守法、诚信、知礼”现代公民道德教育活动，大力弘扬艰苦创业、改革创新、锐意进取的时代精神。积极开展文明城市、文明村镇、文明单位等创建活动，倡导社会文化新风。消除封闭落后的文化习俗，大力推广普及普通话，增强社会的开放性和包容性，吸引各方人才来揭创业发展。

　　优化生态环境。严格控制人口增长，稳定低生育水平，提高人口素质。加强殡葬设施建设，巩固殡改成果。切实加强榕江、练江、龙江、枫江等流域水资源保护和综合治理，重点保护好饮用水源，保障生产用水和群众饮用水安全。加大环保执法力度，强化工业污染控制和城乡环境综合整治。加快市区和县（市）城区污水处理厂、垃圾处理场等环保设施建设，逐步配套完善城乡治污保洁设施，加强城乡污水和垃圾处理。加强资源节约保护，严厉打击盗采矿产资源行为，合理开发土地、森林、矿产、海洋等资源，加快建设绿色揭阳。

　　优化市场环境。加快企业信用建设，推进企业信用分类监管，建立健全信用相关制度、失信惩治机制、信用评级制度，完善社会信用体系。继续整顿规范市场经济秩序，加大治理商业贿赂工作力度，依法严厉打击制假售假、走私贩私、商业欺诈等经济违法犯罪行为。加强食品药品安全监管，保障人民群众身体健康。规范各类中介组织，维护正常的市场秩序。

　　优化社会环境。高度重视公共安全，加强安全生产监督管理，用铁的手腕遏止重特大事故和公共安全事故发生。加强社会矛盾和纠纷排查，认真做好人民调解和信访工作，预防和妥善处置群体性、突发性事件。加大对治安防控的投入，加强社会治安综合治理，依法严厉打击“黄赌毒”、“两抢一盗”、绑架勒索等刑事违法犯罪活动和法轮功邪教组织活动，坚决扫除“六合彩”、“鱼虾蟹”等赌博现象。完善社会治安防控体系，积极创建“和谐文明村”和“平安社区”，打造和谐平安揭阳。

　　十、狠抓自身建设，提高行政效能

　　坚持民主施政。坚决贯彻执行市人大及其常委会的决议、决定，自觉接受市人大及其常委会的监督和政协的民主监督。及时办理人大代表议案、建议和政协委员提案。认真听取各民主党派、工商联、无党派人士和人民团体的意见建议。重视新闻舆论和公众监督。完善基层民主管理制度，推行村务、厂务公开和社区管理事务公开。

　　坚持科学理政。加快政府职能转变，坚决把不该管的事交给企业、市场和中介组织，真正把政府管理职能转到为市场主体服务和创造良好环境上来。健全决策反馈机制，提高行政决策的公众参与度和有效性。充分发挥市政府经济顾问、专家咨询组的作用，完善公众参与、专家论证和政府决策相结合的行政决策机制，规范决策程序，强化决策责任，提高政府科学决策水平和科学理政能力。

坚持依法行政。贯彻实施国务院《全面推进依法行政实施纲要》，大力推行行政执法责任制，依法界定执法职权，科学设定执法岗位，规范行政执法行为。加强行政复议工作，强化行政执法监督，促进政府行为的法制化、规范化。积极开展普法教育，提高公务员队伍的法制意识和依法行政水平。

　　坚持从严治政。切实加强政风建设，大兴求真务实之风，做到重实干、办实事、求实效。深入开展廉政建设和反腐败斗争，健全惩治和预防腐败体系，坚决纠正损害人民群众利益的不正之风。加强督查督办工作，确保政令畅通、令行禁止。加强公务员队伍教育培训和管理，提高办事效率和服务水平，努力建设为民、务实、清廉的公务员队伍。

　　各位代表：揭阳正处在加快发展的新时期，新的目标令人鼓舞，新的形势催人奋进，新的征程任重道远。让我们紧密团结在以胡锦涛同志为总书记的党中央周围，在省委省政府和市委坚强有力的领导下，同心同德，振奋精神，锐意进取，奋力拼搏，不断开创各项工作新局面，为建设富民强市、和谐文明新揭阳而努力奋斗！
